# Organizational Culture as a Determinant of Outcome in Teams: Implications for the Pediatric Cardiac Specialist

**DOI:** 10.1007/s00246-022-03041-5

**Published:** 2022-11-02

**Authors:** Colin J. McMahon, Edward J. Hickey, Lars Nolke, Daniel J. Penny

**Affiliations:** 1 Department of Paediatric Cardiology, Children’s Health Ireland, Crumlin, Dublin 12, Ireland; 2 School of Medicine, University College Dublin, Belfield, Dublin 4, Ireland; 3 School of Health Professions Education, Maastricht University, Maastricht, Netherlands; 4 Department of Cardiothoracic Surgery, Texas Children’s Hospital, Baylor College of Medicine, Houston, TX 77030 USA; 5 Department of Cardiothoracic Surgery, Children’s Health Ireland, Crumlin, Dublin 12, Ireland; 6 Department of Pediatrics, Texas Children’s Hospital, Baylor College of Medicine, Houston, TX 77030 USA

**Keywords:** Congenital heart disease, Culture, Leadership, Organizational behaviour, Organizational culture, Pediatric cardiology, Psychological safety, Teams

## Abstract

Although enormous effort has focussed on how to build an effective culture in the business community, relatively little effort has addressed how to achieve this in the hospital environment, specifically related to the field of congenital heart disease teams. The examination of culture in pediatric cardiac care is particularly important for several key reasons: first, it represents high-stakes medicine, second, there are multiple stakeholders requiring collaboration between cardiologists, surgeons, anaesthesiologists, perfusionists, nursing staff, and allied health care professionals, and finally, both the patient and the family are intimately involved in the care pathway. This review article investigates some of the critical components to building an effective culture, drawing upon similarities in other disciplines, thereby fostering high performance multidisciplinary teams in congenital cardiology care. Strategies to change culture such as Kotter’s model of change are also discussed. High performance teams share one common vital characteristic: *psychological safety* for team members to speak their minds, thereby fostering an open culture, in which creativity can flourish to facilitate major breakthroughs. Adoption of the “Flight Plan” review promotes patient centric care and champions a *psychologically safe* culture.

## Introduction

Survival rates for patients with congenital heart disease is exceedingly high, up to 98% in many international congenital cardiac centres of excellence [1]. Although some effort has been directed towards improving organizational culture in areas of general medicine and nursing, relatively little effort has focused on delineating which elements are critical to building an effective culture in providing congenital cardiology care [2–7]. Similar to an orchestra in which each member must clearly know their role, and play seamlessly with other players, guiding cardiology team members (including surgeons, anaesthesiologists, intensive care staff, nurses and allied healthcare members) to coordinate their efforts can prove elusive [8]. This is because as human beings we are complex, imperfect, and often dysfunctional in complex team settings. Furthermore, this problem may be compounded by ‘type A’ individuals, who are often drawn to the field of congenital heart disease.

Organizational culture is a distinct field of study in the business and management worlds, which addresses the critical components necessary to foster an effective business environment [8]. Culture is “how things get done around here” in such environments [9]. Over the last 2 decades, multiple studies have defined which components are critical to foster a high functioning culture in the business world. This study evaluates some of those critical components necessary to build an effective culture within the medical environment, what we term the “medical organizational culture”. We address how such key components act to shape a successful congenital cardiology service, with its multitude of stakeholders, complexity of patient needs, and requirement for ever evolving innovation. First, we should reflect on the extensive work on organizational culture within the business community and corporate governance worlds before applying the theoretical framework of Hofstede to culture within our own specific domain [10].

## Culture in the Business World

A healthy culture is vitally important to a successful organization or team. Culture is not only integral to the success of any organization, but also the well-being of its employees. Several high-profile public scandals over the last decade have invariably resulted from poorly aligned organizational cultures, e.g. Enron, Worldview, Mid Staffordshire scandal [11–13]. But creating or shaping an effective workplace culture is time consuming, slow, and requires persistence. Most importantly, senior leaders need to be aware of what it is they are trying to shape, which obstacles they can expect to encounter, and how to get there.

## Definitions of Culture

Culture is an enigmatic holistic concept. Typically, when senior business or medical leaders are asked what culture is, they often claim to understand it, but fail to be able to succinctly describe it. Several different definitions encompass how different groups frame their concept of culture. “The way we do things around here” evidenced by the Monkey experiment, highlighted that people blindly follow group behaviour, even when there is no rational explanation underlying it [14]. Edgar Schein wrote extensively on culture, and gave it the widest ranging definition as “A pattern of shared basic assumptions that the group learned as it solved its problems of external adaption and internal integration, that has worked well enough to be considered valid and therefore, to be taught to new members as the correct way to perceive, think and feel in relation to the problems” [15]. Hofstede described it as “the collective programming of the mind, distinguishing the members of one group or category of people from others” [15].

Culture may be context-specific and vary across different cultural jurisdictions [16]. *Mokita* is the concept of culture taken from the Kivila language, spoken by indigenous tribes of Papua New Guinea. Loosely translated it means “The truth we all know but agree not to talk about”. In this context it refers also to the “elephant in the room” e.g. when the culture in the department has degraded and everyone knows but does not say anything, or “polite fiction” when everyone knows the truth but adopts an alternative reality to obviate being embarrassed or ashamed e.g. a senior colleague becoming forgetful at work.

**Fig. 1:**
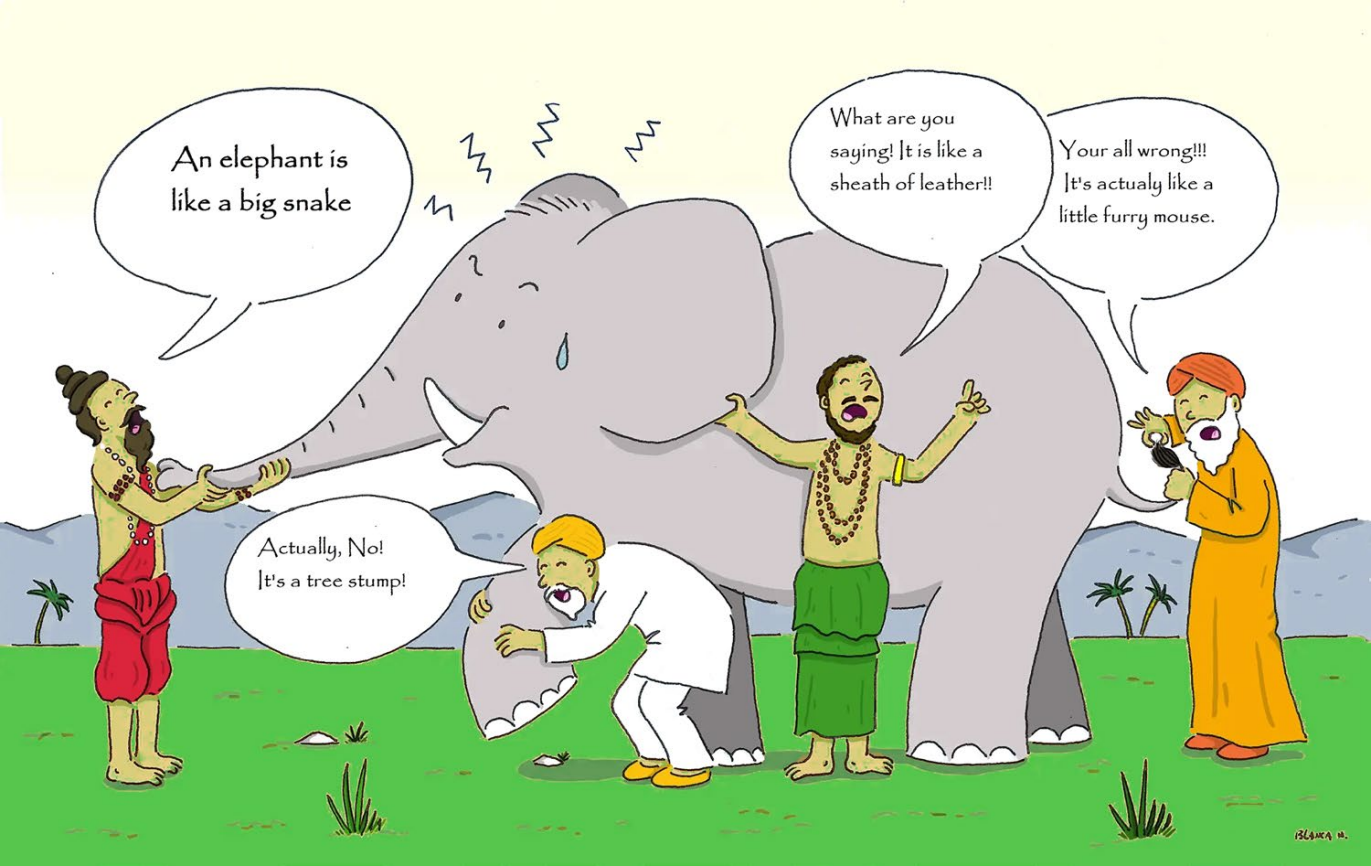
The Sufi Elephant and Blind People Parable

The Sufi parable of the elephant and the blind men is useful, when viewing how people frame culture (Fig. 1). One person may believe it to be a snake, another a tree trunk and another a fan, but it still remains an elephant. This is translatable to congenital cardiology teams. The cardiac surgeon may believe that what they do in the operating room is the core of the entire program, the electrophysiologist, the work in the EP lab, the catheterisation doctor, the interventional procedures and the imaging consultant, work within the echocardiography laboratory. But their frame of reference is too narrow. The core value within the organizational culture is the care of the child and their family.

Hofstede in comparing and contrasting different cultures across nations invented “the cultural onion” [10]. Each culture has several distinct layers, each of which one must peel to reach its core. He described cultural differences being distinct in several ways such as symbols, heroes, rituals and finally values, which reside at the core. Core values may observed through the observation of symbols, heroes and rituals *in action* [17].

## Culture Across Different Nations

**Fig. 2:**
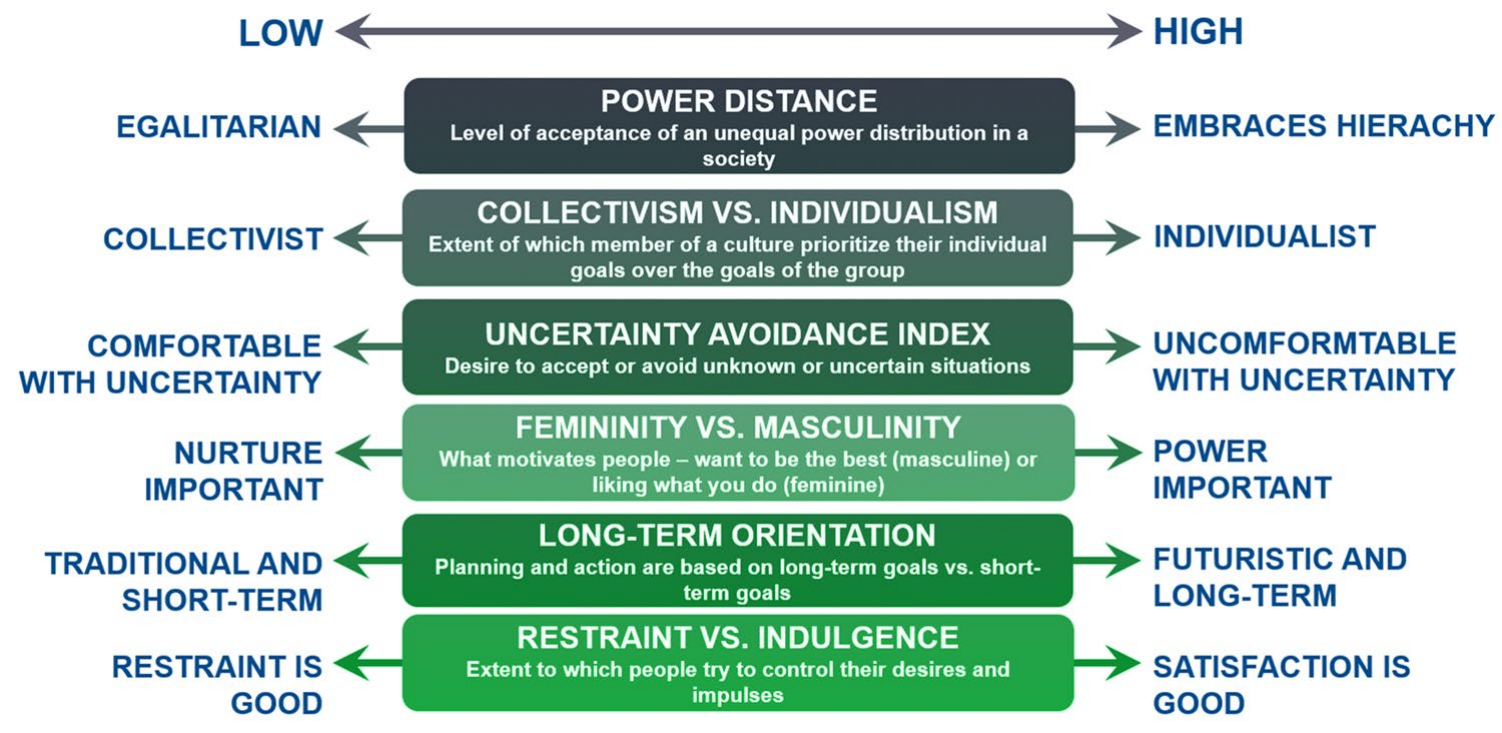
The 6 Components of Culture Framework (Hofstede)

Hofstede in 1980 proposed a framework or theoretical construct, identifying differences in national cultures, based upon 6 different dimensions (Fig. 2) [10]. This framework can also be applied to congenital cardiac units across different countries and cultures. These dimensions include the power distance index, individualism vs. collectivism, uncertainty avoidance, masculinity versus femininity, long-term orientation vs. short-term orientation and indulgence vs. restraint*.* Despite these distinct cultural differences across different nations, what is the actual impact on the culture within the cardiac department?

## The Impact of Culture

*“Culture eats strategy for breakfast”* is a quote often attributed to Peter Drucker, the father of modern management theory. This famous saying effectively highlights that a company may have the best strategy in the world, but if that company's culture is toxic, the strategy is irrelevant, the company will fail [18]. Several famous examples exist of exemplary work cultures in the business world. Herb Kelleher was celebrated as a Chief Executive Officer, who got the culture right at South West Airlines. He built a company where employees remained, even when they were offered higher remuneration packages elsewhere [19]. Employees were hired based upon “fit”, not necessarily the person with the best curriculum vitae. Likewise, all employees were represented on the hiring committee to ensure the “right fit” people were employed. Not surprisingly, pilots and air hostesses helped out unloading luggage, when their help was needed. It was that type of culture where everyone pitched in. Kelleher was so popular with his staff, that they even purchased him a Harley Davidson motorcycle, when he retired from the company. Can you imagine a consultant pediatric cardiology interview where a nurse, junior doctor, parent, ward janitor and lay person were included on the interview board, and not just senior management, or the senior cardiac surgeon. What sort of cultural message would that send to the team?

## Why Does Culture Matter and How Does It Impact on Congenital Heart Programs?

**Fig. 3:**
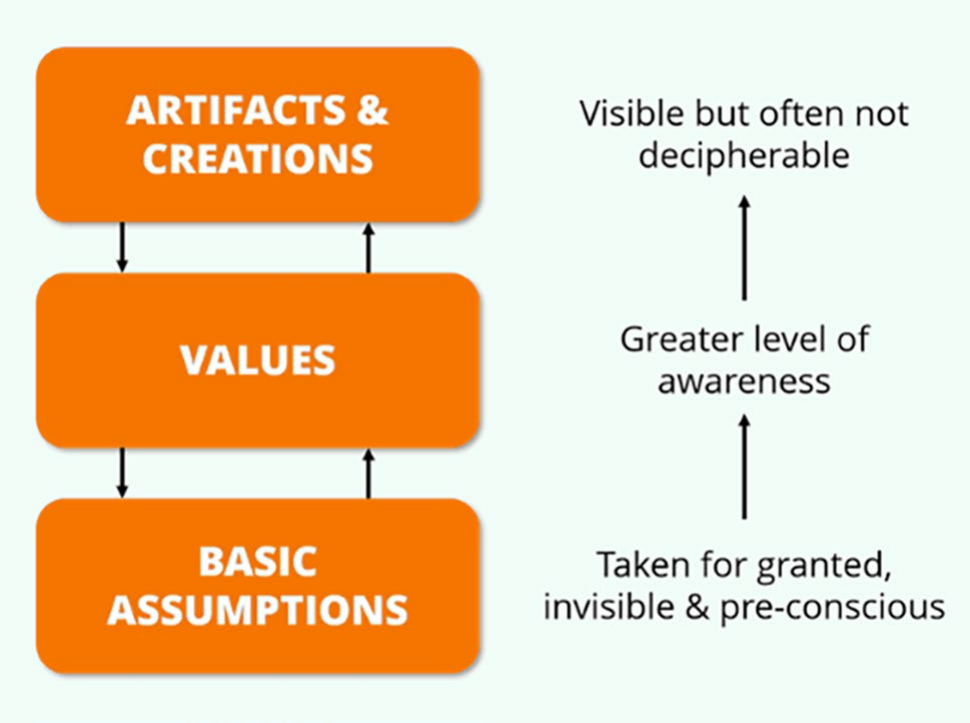
Edgar Schein’s Cultural Framework. Schein outlines three levels of culture including assumptions and beliefs, espoused values, and artifacts

Schein wrote that assumptions and beliefs of employees drives behaviour [20]. Collective behaviour of employees determines results. The results measure performance, and indicate if strategic objectives have been accomplished. Schein outlines three levels of culture including assumptions and beliefs, espoused values, and artifacts (Fig. 3). Culture is not about being nice in this setting, but rather, being purposeful to improve results and outcomes for our patients. Although organizational culture has been described similar to an orchestra, typically with the chief of the department as the conductor, medicine is a little different in that we often have to write the score as we go along. Analogies with improvisational jazz would appear appropriate to our setting, because as leaders we do not know what we are going to play, but if we get the right people together (the band), build a culture in which each of them can thrive and excel, then special things can happen [21]. In improvisational jazz, each band member listens and hears what has gone before, and builds upon that. This is analogous to a culture of listening and hearing colleagues opinions, respecting them (even if opinions differ to our own), and valuing what they have to say. In this work environment we are ‘teaming’ (a verb not a noun), there is only just enough hierarchy. Psychological safety is well-developed so that the cost of making oneself vulnerable is low. As a result, the organization becomes ‘fearless’ and innovation thrives. [22].

A Pubmed Medline search using the search terms ‘organizational culture’ and ‘medicine’ yielded 48,756 articles. Refining the search further to ‘organizational culture’ and ‘congenital heart disease’ yielded only 3 articles. Five of the most important themes discussed included (1) psychological safety (2) trust (3) alignment of values and strategy, (4) open accountable culture and (5) performance measurement.

1. **Psychological Safety**
  Psychological safety has emerged as an important part of a safe culture for teams, and it effectively means being able to show, and employ one's own self without fear of negative consequences of self-image, status or career [23]. It can be defined as a shared belief that the team is safe for interpersonal risk taking. In psychologically safe teams, members feel accepted and respected. It is one of the most studied enabling conditions in group dynamics and team learning research.
  Clark [24] has contributed to the concept of psychological safety outlining the four stages of psychological safety as “a condition in which human beings feel included, safe to learn, safe to contribute, and safe to challenge the status quo—all without fear of being embarrassed, marginalized, or punished in some way.”
  Psychological safety benefits teams in several different ways including increased likelihood for successful process innovation, increased team member learning from mistakes, better team engagement, and increased team innovation. Leaders play a critical role in augmenting team members' psychological safety, through the use of participatory management, and inclusive management. Two specific aspects of team culture that improve its psychological safety include a clear team structure where members understand what is expected of them, and a strong collaborative relationship between team members.
  Amy Edmondson highlights the importance of psychological safety to facilitate people innovating and being purposeful [22]. People constantly manage personal risk at work, and either consciously or subconsciously, avoid openly sharing their ideas or concerns. If team members cannot be open about what they think is wrong, how can they improve the culture, the workplace, or the patient care? We have to risk vulnerability to admit there are problems in our institution, department or team. One effective tool to facilitate this is ‘Open Space Technology’, where all the team members add ‘post-its’ to a Board. Groups of team members then form around common problems, and volunteer to find a solution together to the problem. This is the ultimate democratization of change, and empowers people to be that change. The team becomes responsible for their own success, not the conductor.
  Leaders of teams play a critical role in shaping a climate of psychological safety. Leaders who only welcome positive news create an environment of fear, which prevents them from hearing the truth. This lack of psychological safety can give the illusion of success, but this invariably turns to failure. Excessive confidence in authority can pose a risk factor to psychological safety. A culture of silence is a dangerous culture [22].
2. **Trust**
  “The first responsibility of a leader is to define reality. The last is to say thank you. In between the two, the leader must become a servant.” [25].
  Trust is consistently reported as one of the top requirements for a high performance culture. Employees in high-trust organizations are more productive, have greater energy at work, collaborate better with colleagues, and are retained longer than those who work at low-trust companies. They also suffer less from chronic stress and are happier with their lives, both of which fuel stronger work performance. High-trust environments hold people accountable, but they do not micromanage them [26]. Eight strategies have emerged which foster trust in the workplace: recognise excellence, induce challenge stress, give people direction in how they work, enable job crafting, share information broadly, intentionally build relationships, facilitate personal growth of staff, and demonstrate vulnerability [26].
  By surveying employees about the extent to which firms practiced these eight behaviours, level of trust for each organization was calculated [26]. The U.S. average for organizational trust was 70%. Forty-seven percent of respondents worked in organizations where trust was below average, with one firm scoring only 15%. Overall, companies scored lowest on recognizing excellence and sharing information (67% and 68%, respectively). Companies could enhance trust by improving in these two areas, even if they didn’t improve in the other six [26]. The effect of trust on self-reported work performance is dramatic with top quartile employees reporting they had 106% more energy and were 76% more engaged with work compared with respondents whose firms were in the bottom quartile. Employees in high-trust companies enjoyed their jobs more, were more aligned with their companies’ purpose, and reported a greater sense of accomplishment [26].
  Communication among teams is critical to building trust [27]. This in turn can promote synchronicity among the healthcare team, breakdown resistance to change, and lead to the development of a safety culture at work [28–31]. Stephen Covey talks about the fact that “you can only innovate at the speed of trust” [32].
  Patrick Lencioni author of “The Five Dysfunctions of a Team” talks about overcoming these to become a healthy organization [33]. The first is absence of trust, followed by fear of conflict, lack of commitment, avoiding accountability, and inattention to results. Without a strong foundation of trust, none of these can be achieved.
3. **Alignment of Values and Strategy**
  In the business world, attitudes and core values have been shown to directly impact upon behaviour, which in turn, translates directly to outcomes (Fig. 3). If the core values and attitudes are dysfunctional or misaligned, this will translate to aberrant behaviour and potentially poor results. e.g. if the hospital is a private practice institution and performs tests which are not essential, the core values of that unit is compromised, and behaviour is deviant with a view towards generating revenue, and not looking after the best interests of the patient. Likewise, if there is a culture of bullying in the department, which is tolerated, this translates to a culture of fear and poor outcomes. So the core values and attitudes of leaders within the department are critical in directing the behaviours and outcomes for that culture. Similarly, if there is a lack of transparency and open dialogue when problems arise, this can lead to seriously compromised behaviour, and devastating outcomes.
  The Bristol enquiry highlighted a culture of silence to the high mortality rates for children undergoing cardiac surgery (between 30–35 children died between 1991 and 1995) [34]. Approximately one third of children who underwent open heart surgery received less than satisfactory care. The inquiry reported a flawed system of care with poor teamwork between professionals, “too much power in too few hands,” and surgeons lacking insight, to see that they were failing, and that they should cease operating [34]. According to the inquiry report, there was a “club culture” with insiders and outsiders, the management side was punitive, with an environment which did not promote psychological safety [34]. Further analysis showed broader inadequacies at every point, from referral to diagnosis, surgery, and intensive care. The physical set-up was dangerous with surgeons on one site and pediatric cardiologists at the children’s hospital. Ian Kennedy who chaired the enquiry reported “To a very great extent, the flaws and failures of Bristol were within the hospital, its *organisation and culture* and within the wider NHS as it was at the time.” [34] When a whistle blower, Stephen Bolsin, finally came forward, the dysfunctional culture had persisted for a significant time. How many lives could have been saved if staff members felt psychologically safe to speak up earlier, when there were clearly problems with surgical outcomes?
4. **Open Accountable Culture**
  In an open accountable culture, applying Hofstedes 6 criteria approach [15], one might expect a low power distribution (a flat democratic rather than a rigid hierarchical structure), a collectivist approach (the “we” over the “I”), tolerance of uncertainty and ambiguity, adoption of feminine “preference for cooperation, modesty, caring for the weak and quality of life” rather than purely masculine “competition and achievement orientation”, longer term orientation embracing adaptation and circumstantial, pragmatic problem-solving as a necessity, and finally a restrained “control of gratification of needs of medical staff.” Staff treating each other with respect, and with a sense of trust, is crucial to nurturing such a culture. Likewise, honesty when there are problems, in terms of difficult colleagues [35] or poor surgical outcomes is fundamental to such an open culture [36]. Calling out bullying, or lack of respect for diversity, and inclusion would also signal an open accountable culture [37]. Lastly, an open culture must be one, which is open to change or evolution, and not rigid or fixed. Emergent change is often bottom up, rather than top down change, and is challenging to implement in cultures which are rigid or fixed. Leadership and trust are fundamental to such change, and micro-moves are possible with a background of effective teams, clear vision, stakeholder engagement, adequate resources, clear communication, with a functional hierarchy (parallel hierarchy) and professional bureaucracy [38].
5. **Performance Measurement**
  Measurement of team, and also individual performance, is critically important. Deming argues “In God we trust, all others bring data” [39]. Performance measurement systems are utilised for monitoring, attention focusing, strategic decision-making and legitimization [40, 41]. Key performance indicators are also used to provide feedback, and to clearly outline expectations with employees/clinicians. John Doerr talks about the importance of “Measure what Matters” [42]. Big Data is becoming critically important in this realm, as it allows the team visualise the big picture, and extract actionable insights, which can have dramatic impacts on patient care and outcomes.

## The Importance of Leadership?

What qualities do leaders need to exhibit if they wish to foster a quality culture? Perhaps, our first impression of leadership is the caricature alpha male, making all the decisions, sitting at the top of the multidisciplinary team meeting, owning the conversation, beating down any dissident voices with his booming voice, and imprinting his will on the rest of the team, who invariably feel superfluous to the whole process. Not surprisingly, this is not the most effective form of leadership and thankfully, in the main, is an icon of the past. Rather, Collins in his hierarchical model of leadership describes the ‘Level 5 Leader’ who displays a somewhat paradoxical combination of modesty and drive, mixed with an unwavering resolve to do whatever it takes to achieve that end, funnelling personal ambition into company or team success [21]. Similar to the conductor, Benjamin Zander suggests that his(her) power is to make other people powerful, to awaken the possibility of greatness in them, to build trust by delegating responsibility to them for their work, and give them the freedom to shine (Benjamin Zander, TED 2008). Like Carlos Kleiber, the conductor or chief’s job is to create the environment for the person to thrive, to be accessible, approachable, supportive and by their side as they reach their potential. This may sound rather woolly, but on reflection, we all recognise good and toxic culture departments we worked in, or visited, over the years, if we are honest with ourselves.

Leadership should permeate throughout the whole team, and our mindset should evolve in how each of us view leadership. The janitor on the ward is as important as the chief cardiologist. She or he is protecting patients through their capacity to maintain a clean healthy environment. No operation goes ahead if the theatre isn’t sterile. When John F. Kennedy visited NASA, and asked a janitor what he was doing, the man replied “ I am putting a man on the moon, Mr. President.”

## How to Implement Change Within High Performance Congenital Cardiology Teams?

John Kotter published a landmark eight-step change model in ‘Leading Change’ in 1995 [43]. This work, building upon previous work by Kurt Lewin, set out 8 key steps in the change process. It also emphasised that avoiding any step may result in the entire initiative failing. If we imagine a crisis in the congenital cardiology unit e.g. mortality figures for the arterial switch operation suddenly exceed 10% or an employee leaving the department claiming a culture of bullying, how would we implement change in that culture?

Let us consider an imaginary scenario: Three successive children with d-transposition of the great arteries die within four weeks of surgery over a three-month period at a tertiary care centre. A review of the previous five years finds that there was one death among 50 other transposition patients (2% mortality). The surgeon meets with you concerned about these outcomes. How can we apply Kotter’s leading change framework to address this alarming problem?

### Step 1. Create a Sense of Urgency

A dramatic increase in mortality rates from a specific surgery (e.g. arterial switch operation) should automatically generate a sense of urgency in the entire department. Kotter argues that 75% of the team need to be in agreement for the change process to improve. Open communication of the increased mortality of these children is paramount. This is an urgent problem and needs to be immediately addressed.

### Step 2. Form a Powerful Coalition

**Fig. 4:**
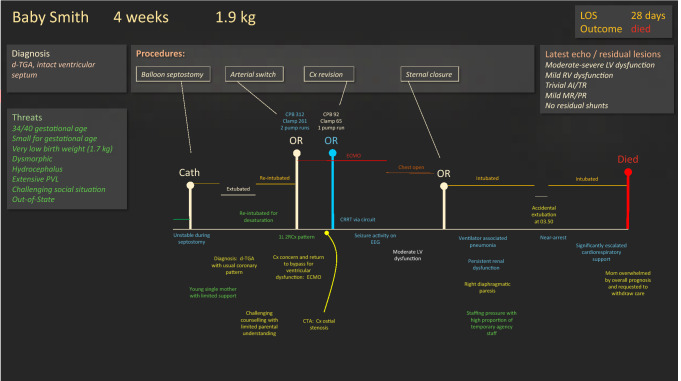
Flight Plan. A flight plan (simulated) depicting the perioperative journey of a neonate with transposition of the great arteries (d-TGA) with intact ventricular septum. Many *threats* (green) surrounded the child’s case, which contributed to *errors* (yellow) and *unintended states* (blue). Several chains of threats/errors/unintended states led to progressive loss of safety margins that ultimately contributed to the family’s decision to withdraw care: (a) Prematurity and low birthweight contributed to technical challenges for both septostomy and arterial switch including contributing to challenges with coronary button implantation, which led to extended cardiopulmonary bypass runs, coronary revision surgery and ECMO support with significantly delayed sternal closure and sepsis/pneumonia and renal failure risk (b) Diagnostic errors missed the atypical coronary pattern and contributed to the errors in management of button re-implantation and subsequent chain outlined in (a) and residual lesion of moderate-severe ventricular dysfunction (c) Small patient size and multiple surgical interventions increased the risk of phrenic nerve injury and therefore reduced respiratory reserve (d) Latent staffing threats may have contributed to the risk of, and impact of, accidental extubation in the middle of the night, which resulted in near cardiorespiratory arrest and significantly escalated support with guarded prognosis. (e) Prematurity and low birthweight contributed to the extensive periventricular leukomalacia and hydrocephalus and subsequent neurologic vulnerability and concerning long-term prognosis, exacerbated by the added insult of ECMO and near-arrest at the time of accidental extubation. (e) The social circumstances of a very young mother with limited support, remote from her family support contributed to counselling challenges and difficulties digesting the clinical picture and overall prognosis. The flight plan for this child’s journey reveals several areas for program improvement including: (1) diagnostic imaging, (2) 360° pre-operative prognostication, (3) education and counselling, (4) staffing models and (5) support for especially vulnerable parents

Enlisting all the key stakeholders is critical, not just the cardiothoracic surgical team. A coalition of key stakeholders, including cardiologists, anesthesiologists, intensivists, nursing staff, and perfusionists should be formed to analyze the reasons for increased mortality. The program might consider a pause in arterial switch procedures until the cause can be clearly identified and resolved. A stepwise critical analysis (Flight plan) of every step for each of the patients, beginning at the time of presentation to the unit, should be performed until potential contributory factors can be identified for each mortality (Fig. 4).

### Step 3. Create a Vision for Change

Clearly highlighting the specific problem is important so each team member knows what is involved. The vision should be simple and understandable but also inspirational to have optimal impact e.g. it could be developing an overall pathway for management of transposition patients from early foetal diagnosis to rapid transfer to the cardiac department (co-location with maternity services), guidelines for management of cyanotic patient, preoperative detection of coronary arterial pattern, standardised intraoperative procedure, retraining of surgical skills where needed [31] and a cohesive intensive care unit approach.

De Leval, in a landmark paper, detailed the very honest and open assessment of mortalities among 102 arterial switch patients [31]. A detailed factor analysis was undertaken, but after the death of the 68th patient in the group, the surgeon recognised that in about half of the cases, no obvious variable could be identified. He then recognised the need to undertake a period of retraining at a low-risk centre, which reduced the mortality associated with the procedure.

### Step 4. Communicate the Vision

The vision needs to be communicated throughout the department using the coalition of clinical team members. The vision needs to be continuously communicated as there may be competing messages being circulated. In our scenario, some individuals may believe that this is a cluster and surgery should continue, but communicating the need to identify factors, which may have led to the outcome is critical, before further arterial switch surgeries proceed.

### Step 5. Remove Obstacles

Whether it is individuals, traditional ways of doing things, or competing interests, there are often barriers to implementing the change. If the obstacle is delivery of transposition babies in distant centres then that obstacle must be removed, by improved antenatal detection, and local delivery, allowing safer preoperative stabilisation. Individuals unable to function in the team, may need to be deployed to a different area of work.

### Step 6. Create Short-Term Wins

Given the time involved in implementing successful change, creating some early wins is important to demonstrate the benefits of the new process. This may help with motivation for the team to persist with the change process. For example, in the transposition scenario, optimising the process through improved fetal detection, day time appropriate delivery in adjacent maternity services, quicker efficient transfer to the cardiac unit, and efficient stabilisation and optimisation of the child prior to surgery all create short-term wins with a more stable child. Many of these changes can be won simply through improved organisation and coordination efforts.

### Step 7. Build on Change

Persisting until the change process is completed can be difficult and there is a tendency for complacency to settle in or people to fall into old habits. Kotter argues the importance of cementing the change long after it has been implemented. Developing a culture of quality improvement and continuous change in the department is important. Implementing a mechanism of continuous monitoring of patient outcomes, in terms of morbidity and mortality for all cardiac surgeries not just arterial switch, would represent building on change in this scenario.

### Step 8. Anchor the Change in the Culture

Altering the behaviour of individual team members may not be sufficient to bring about a lasting culture change within the cardiology department. These changes should become part of the core of the organisation to have a permanent effect. Keeping stakeholders on board, encouraging new employees to adopt the changes and recognising individuals who adopt the change all promote a permanent change in culture. In our transposition scenario, empowering all stakeholders to feel psychologically safe, to highlight worries or concerns about patient management at any point in the future, would highlight an anchoring of that change in culture.

## Incorporating the 8 Steps into a “Flight Plan”

The safety culture of commercial aviation and other high-stakes industries has evolved not just from analysing disasters, but largely from learning and studying patterns in routine, live flight. Fly-on-the-wall *line operating safety audits* (LOSAs) have been an integral part of commercial aviation for over 30 years [44]. *Errors* (human action or inaction that leads to a reduction of safety margin) are recognized to be common, ubiquitous and inevitable, and are usually prompted by ambient *threats*. To paraphrase the Australian Civil Aviation Authority, “a threat is anything that takes you away from the ideal day.” They come *at* the crew and are the risk factors for errors occurring; in medicine, they include patient risk factors but also *latent* threats, such as operational conditions, culture, management structure or aspects of training, which indirectly lead to greater risk of error. The importance of latent threats lies in the fact that unless they are addressed, it is highly likely that errors will recur. Errors can be effectively mitigated if recognized promptly and managed appropriately. However, if not effectively mitigated, a chain can emerge of error and unintended state leading to progressive loss of safety margins [44].

The interplay between threats and errors—and their propagation into chains—is known to be similar in high-stakes clinical medicine and is best understood through a graphic representation of a patient’s course [45]. The so-called *flight plan* was devised at Sick Kids, Toronto and depicts of a child’s peri-operative journey with unexpected clinical deviations and escalations in risk easily conveyed—it is analogous in many ways to LOSA in a commercial airline cockpit. Root causes (*upstream sentinel errors* and the threats that triggered them*)* and the impact of additional *amplifying errors* are far easier to decipher than in conventional morbidity and mortality review sessions [46].

At Sick Kids (Toronto, Canada) and Texas Children’s Hospital (Houston, Texas) Heart Centers, patients’ flight plans are reviewed in weekly, department-wide quality improvement sessions [44]. The success of such sessions is dependent upon leaders endorsing an atmosphere of psychological safety and fostering open discussion that includes demonstrating modesty and vulnerability. Flight plan review of patients (mock example in Fig. 4) can yield a broad array of benefits: (a) reinforcing individual accountability, (b) identifying near-misses, (c) highlighting systemic and latent threats, (d) reinforcing the importance of team cohesion, (e) increasing visibility and optics, (f) promoting education, (g) providing a clinical safety net for on-going patient concerns and follow-up, and finally (h) as a department-wide session, implementation of strategies or action-items can be extremely rapid [44].

## Conclusions

In conclusion, we have reviewed the definition of culture, how it may appear to different stakeholders and its importance in terms of patient outcomes for the pediatric/congenital cardiac specialist. We have outlined five core components to culture, including *psychological safety*, trust, alignment of values and strategy, open accountable culture, and performance measurement. Kotter’s eight-step change program, with some adaptation to the medical environment, provides a useful framework in the context of change within cardiac programs.

Adoption of the “Flight Plan” review incorporates several of these steps. Although further work is required to examine these concepts in greater detail, it is clear that a positive culture is purposeful in achieving excellent outcomes for our patients, and it derives primarily from building an environment of *psychological safety* for each and every member of the cardiac team.

## Abbreviations

LOS: Length of stay
PVL: Periventricular leukomalacia
Cx: Circumflex coronary artery
ECMO: Extracorporeal membrane oxygenation
CTA: CT angiogram
LV: Left ventricle
RV: Right ventricle
AI: Aortic insufficiency
TR: Tricuspid regurgitation
MR: Mitral regurgitation
TR: Tricuspid regurgitation
